# Hearing with One Ear: Consequences and Treatments for Profound Unilateral Hearing Loss

**DOI:** 10.3390/jcm9041010

**Published:** 2020-04-03

**Authors:** Hillary A. Snapp, Sebastian A. Ausili

**Affiliations:** Department of Otolaryngology, University of Miami, 1120 NW 14th Street, 5th Floor Miami, FL 33136, USA; s.ausili@miami.edu

**Keywords:** unilateral hearing loss, single-sided deafness, monaural, spatial hearing, cochlear implant

## Abstract

There is an increasing global recognition of the negative impact of hearing loss, and its association to many chronic health conditions. The deficits and disabilities associated with profound unilateral hearing loss, however, continue to be under-recognized and lack public awareness. Profound unilateral hearing loss significantly impairs spatial hearing abilities, which is reliant on the complex interaction of monaural and binaural hearing cues. Unilaterally deafened listeners lose access to critical binaural hearing cues. Consequently, this leads to a reduced ability to understand speech in competing noise and to localize sounds. The functional deficits of profound unilateral hearing loss have a substantial impact on socialization, learning and work productivity. In recognition of this, rehabilitative solutions such as the rerouting of signal and hearing implants are on the rise. This review focuses on the latest insights into the deficits of profound unilateral hearing impairment, and current treatment approaches.

## 1. Introduction

Hearing loss is the third leading cause of disability globally [1], with approximately 466 million people living with disabling hearing loss around the world [2]. Hearing loss is associated with increased mortality and has widespread effects on overall health, including links to cognitive decline, cardiovascular disease, depression, sleep disorders and impaired socialization [3,4,5,6,7,8]. The societal impact is high. The estimated costs of this global health burden exceeds 750 billion U.S. dollars annually, and is largely due to higher unemployment rates and lost work productivity [9]. These estimates, however, do not take into account the proportion of adults with undiagnosed or unaddressed hearing loss, such as those with unilateral hearing impairment [10,11]. Disabling hearing loss has long been associated with bilateral age-related changes. However, disability and handicap in those with unilateral hearing loss have been shown to match or exceed those that are reported in individuals with bilateral hearing loss, despite hearing with only one normally functioning ear [12,13]. Accumulating evidence points to the loss of spatial perception as the driving factor in functional disability for those with unilateral hearing loss. This review explores the consequences of unilateral profound deafness and reviews treatment and rehabilitative strategies.

Profound unilateral sensorineural hearing loss, often termed single-sided deafness (SSD), refers to clinically-unaidable hearing, as defined by severe-to-profound hearing thresholds with a poor word recognition ability [14]. Acquired unilateral hearing loss occurs in 12–27 per 1,000,000 persons annually [15]. The etiology of SSD is widespread, including such pathologies as cochleovestibular abnormalities, temporal bone trauma, Meniere’s disease, vestibular schwannoma, vascular ischemia, autoimmune disorders and infections, although it is commonly idiopathic in nature. Often, this loss can be sudden in onset, leaving the patient extremely debilitated [12,16]. Despite this, prevailing misperceptions that one normal hearing ear is sufficient for daily communication persists. Long overlooked are the deficits and disability associated with SSD, which have substantial but differential impacts on the affected. Unlike other paired systems, such as vision, where the impact of unilateral impairment is readily acknowledged, hearing is subject to an invisibility factor, where the disability itself is less overt and subsequently underappreciated. As such, the impact of SSD is often underestimated. Increased effort is required to compensate for unilateral hearing loss in complex listening environments [17]. Over time, such additional stressors result in auditory fatigue [13,16,18,19] and reduced performance at work [9]. The increased hearing handicap in this group is most strongly linked to deficits in spatial perception.

## 2. Spatial Hearing

Spatial perception is multisensory and multifaceted. The auditory system plays a particularly important role, helping to map where we are in space by continuously sensing auditory events. The constructs of auditory space are complex, depending on our interaction with signals that are dynamically changing in terms of frequency spectrum, level and time. The more complex the listening environment, the more these signals interact and overlap. Therefore, hearing is not just about sound detection or awareness, but also about managing these complex interactions to provide meaning to those signals. Our auditory system is quite sophisticated in wholly managing the acoustic information presented to our ears in dynamically changing environments. Human listeners are able to rapidly process this information to identify and orient to acoustic stimuli, selectively attending to signals of importance while suppressing competing signals. This is all seemingly accomplished quite simply and effortlessly across numerous everyday environments, while allowing listeners to be highly functional.

**Figure 1:**
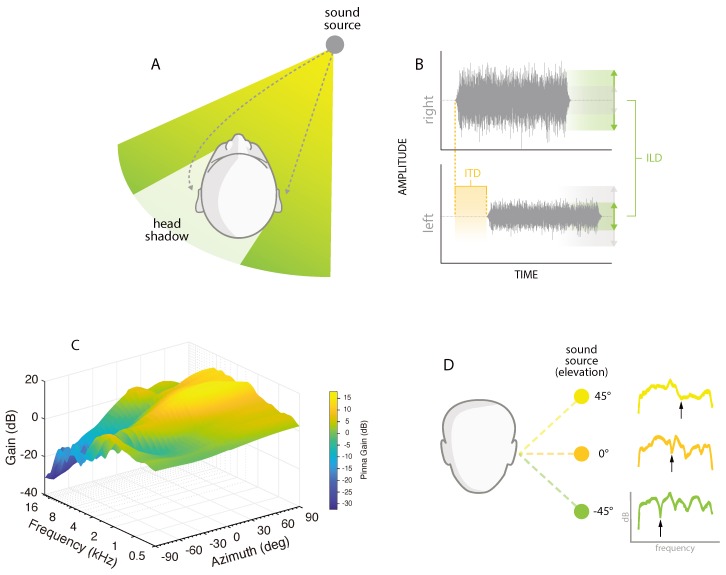
(**A**) An illustration of the acoustic head-shadow and the principle of time-delay between the two ears, dependent on the sound source. (**B**) Right and left temporal signals arriving from a right-leading sound source location. The ILD and ITD are illustrated on the amplitude and temporal domains, respectively. (**C**) The frequency and azimuth dependencies of the head and pinnae filter are shown. As the sound source moves from the hearing side (positive azimuth angles) to the deaf side (negative azimuth angles), the acoustic barrier created by the head attenuates high frequency signals contralateral to the source. Pinna gains are also observed for some frequencies at the ear ipsilateral to the source. (**D**) An illustration of the monaural spectral-pinnae cues as a function of the vertical position (elevation) of the sound source. The black arrows indicate the position-dependent frequency notch.

Spatial hearing is dependent on the processing of monaural and binaural hearing cues (Figure 1A,B). While monaural spectral-shape cues provide important information regarding elevation and contribute to our ability to determine the distance of a sound source, binaural hearing cues play a much larger role in spatial hearing abilities (Figure 1D). The integration of acoustic information from both ears is essential for spatial hearing, and serves to provide critical information for speech processing, localization, the segregation of auditory streams and the perception of fused sounds. Binaural hearing gives rise to a wide array of auditory phenomena due to the integration and processing of differences in arrival time and intensity between the signals at the two ears. The interaural timing difference (ITD) is the difference in arrival time for a stimulus to reach both ears (Figure 1D); it is greatest for low frequency signals below 1000 Hz. Sounds presented directly to the front of a listener have an ITD of 0 μs, increasing in time as the signal moves laterally in the horizontal plane, with the largest ITD occurring for signals presented ±90° azimuth, reaching ~600 μs [20,21]. Due to the extreme binaural sensitivity of this cue, when a sound is presented to a listener, the ear closest to the signal of interest will detect that sound before the ear farthest from the signal. Likewise, the interaural level difference (ILD), or the difference in the intensity of a stimulus reaching both ears, dictates that the ear closer to a stimulus will receive a more intense signal compared to the contralateral ear [22] (Figure 1B). As the signal deviates away from 0° azimuth in either direction, the ILD increases. As with ITDs, ILDs are a frequency-dependent cue, with level differences increasing as a function of frequency [21], reaching values of 20 dB of attenuation or more. This is due to the acoustic shadow created by the head [23]. The head acts as a physical barrier to sounds, resulting in an attenuation of the signal in the ear not directed at the source. The acoustic shadowing of the head varies according to the frequency and position of the signal (Figure 1C).

Sounds arriving at the ears from different locations in space allow listeners to take advantage of spatially separated sounds to improve the signal-to-noise ratio (SNR). For example, the ear closest to the signal benefits from the shadow created by the head to block noise that would otherwise mask the signal. Furthermore, the integration of the input from the two ears results in a summation of the signals leading to perceived enhancement. In addition to boosting the target signal, a reduction of competing noise occurs with binaural hearing [24]. This is known as the squelch effect, where the processing mechanism takes advantages of amplitude and phase differences of the inputs as they arrive at the two ears to suppress competing noise. Collectively, these binaural processes provide listeners with a 4–10 dB benefit in processing speech in complex environments [25].

## 3. Unilateral Profound Deafness

**Figure 2:**
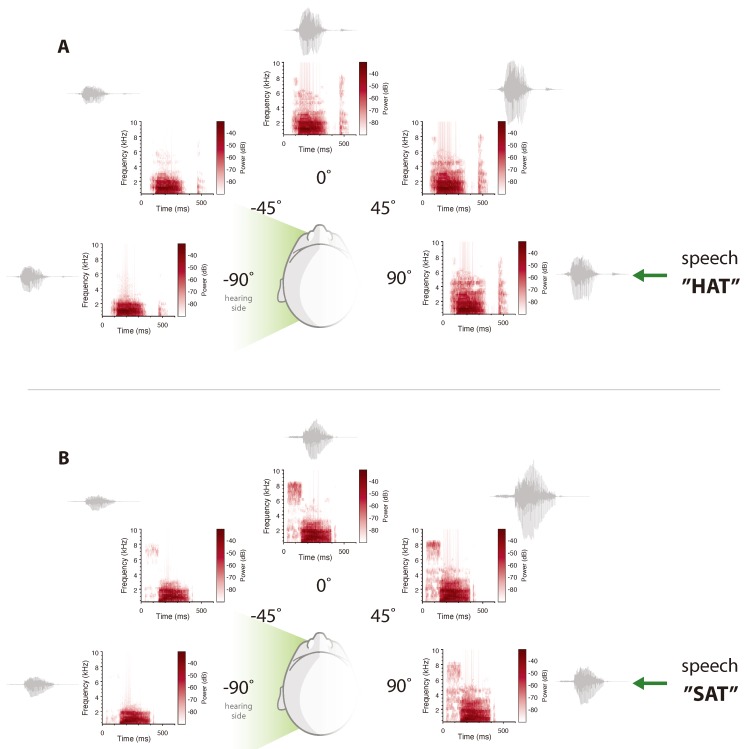
Comparison of the head-shadow and pinnae filtering for the words (**A**) *”Hat”* and (**B**) *“Sat”* when presented at the deaf side. Spectrograms and sound waveforms (in gray) of the speech stimuli are shown for five corresponding azimuth positions. High frequency speech components are attenuated on the hearing side, erasing the unique informational marks that differentiate the words from each other.

These essential interaural hearing cues are unavailable to those with SSD. The reduced ability to discriminate ILDs and ITDs results in primary deficits in speech understanding in noise and localization [26,27]. As monaural listeners, they are left to rely on the normal hearing ear to process all of the incoming acoustic information, thereby losing the ability to segregate spatially separated streams of sound [28,29] or take advantage of spatially separated signals in complex listening environments [28,30]. This is further complicated by reduced access to high frequency speech cues as a result of the acoustic head-shadow [31]. For monaural listeners, access to sound at the normal hearing ear is both frequency- and direction-dependent. As can be observed in Figure 1C, low frequency information can be well detected even when directed at the deafened ear. Low frequency signals are characterized by long wavelengths, which allow them to easily wrap around the head to stimulate the normal hearing ear. Conversely, high-frequency pinna cues are diffracted by the head, making them virtually undetectable when presented at the side of the deafened ear. The auditory system’s ability to adapt to the lack of binaural cues to preserve spatial hearing abilities as much as possible is remarkable. This process is achieved by reweighting the available cues, exploiting the location-dependent monaural cues [32]. However, in a normal every-day acoustic scenario, sounds are constantly changing in level, location and frequency, which makes it impossible to have accurate spatial hearing based only on monaural cues. Losses in gain for deaf-ear listening progressively increase as a function of frequency, reducing access to high-frequency phonemes that give rise to speech intelligibility and discrimination [33]. The resulting outcome is a disruption in speech perception, which is further amplified in the presence of competing noise [33,34]. Specifically, the parts of speech that provide listeners with the ability to distinguish one word from another are inconsistently available to monaural listeners. This can be observed in Figure 2A,B, where the acoustic interactions between the filter of the head and pinnae, and differing speech stimuli are illustrated. The spectrograms represent the frequency components over time on each corresponding position of the horizontal space. The waveforms help to visualize the overall loudness effect on the signal (i.e., changes in amplitudes) caused by the sound interaction with the head and pinnae. Here, noticeable changes are observed in the spectrum of the words for direct signals (+90°, deaf side) compared to those in the acoustic head-shadow (−90°, hearing side). The acoustic construct of the “s” and “h” phonemes is fundamentally different (+90°). The available acoustic information not only makes these two words intelligible but also unique and, therefore, distinguishable. The head-shadow filter is evident in Figure 2A,B at −45° and −90°, where there is a decrease in the overall level and attenuation of the high frequency components of the speech, resulting in an indistinguishable speech construct at the hearing side. The frequency information on the hearing side clearly shows that each word conserves the main speech formants, but has lost the unique informational marks that makes it singular from the others. Since this is the only information available on the functional side, the hearing brain will not be able to correctly differentiate the presented words, leading to difficulties in contextual interpretations. So, while the monaural listener may detect or hear the signal, the ambiguity of the speech sounds diminishes their ability to extract meaning from that signal. This directly contributes to the high levels of handicap and disability reported in this population [12,16,17].

**Figure 3:**
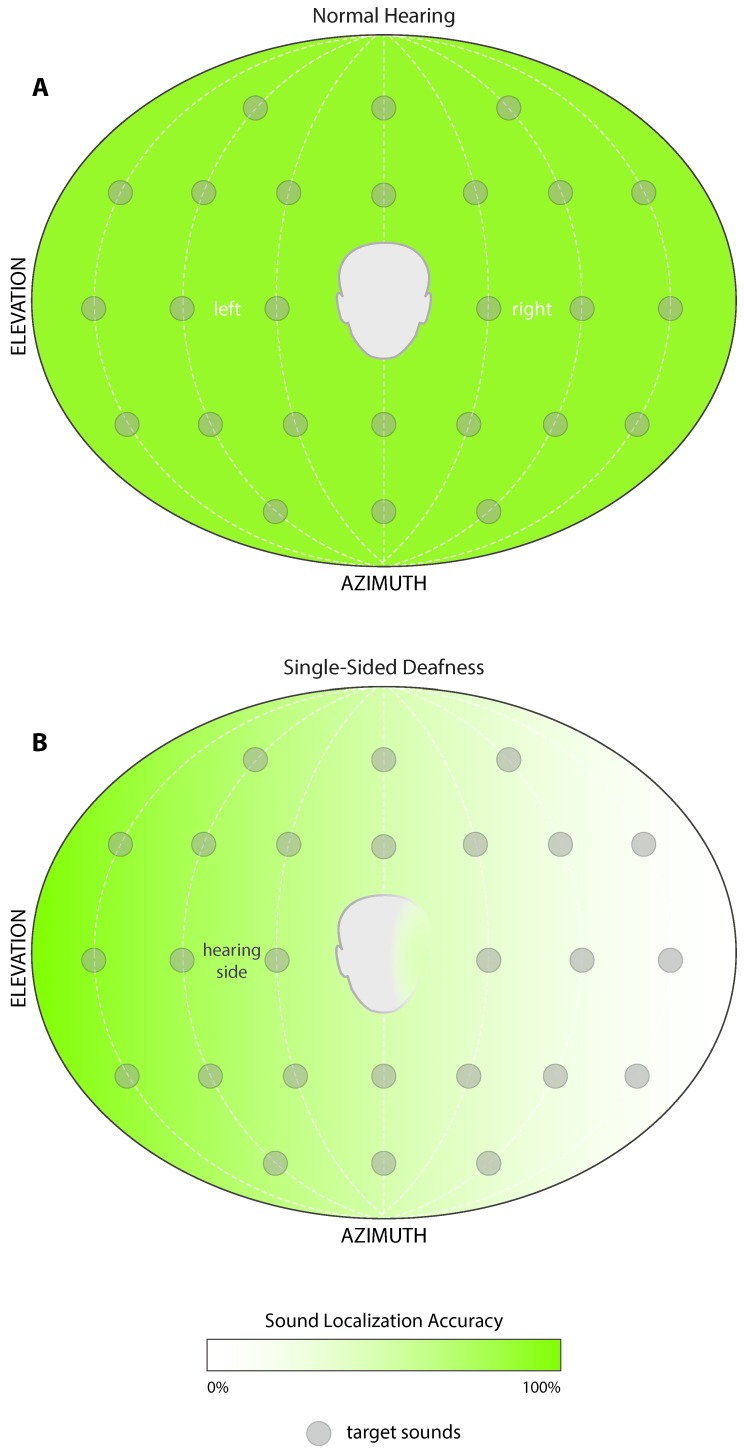
An illustration of spatial hearing abilities for (**A**) normal hearing and (**B**) single-sided deaf listeners. Normal hearing listeners localize target sounds accurately and precisely along the horizontal (azimuth) and vertical (elevation) planes. Monaural hearing listeners, instead, can localize target sounds mainly in the hemifield of the hearing ear, with impaired performance on the contralateral deaf side.

Normal hearing listeners are highly accurate in their ability to localize sound in space, whereas this is largely disrupted in monaural listeners [35,36] due to the lack of available ITD and ILD cues. This hinders the ability of a monaural listener to accurately and rapidly orient themselves to signals of interest or importance. A number of functional deficits arise from the inability to localize, such as issues regarding safety as they navigate the world around them, the inability to locate the talker, target confusions in multi-talker situations, and an overall sense of uncertainty in complex listening environments. However, monaural listeners can adapt to use spectral pinna cues of the normal hearing ear to quell some of the consequential spatial deficits [37,38,39] that arise from the loss of binaural hearing. Figure 1B displays the amplification of high frequency cues that occurs when directed from the better hearing ear. The pinna provides a gain increase of approximately 15 dB for 2–4 kHz. In addition to contributing to speech discrimination, high frequency spectral cues are important for localizing sound in elevation and determining if a signal is in front of or behind a listener [22]. Figure 3A illustrates the high accuracy target identification ability of a normal hearing listener, and how the loss of ITDs and ILDs results in a primary deficit for the location of signals in azimuth. Figure 3B indicates how spectral cues contribute to good localization accuracy for signals presented toward the hearing side, but spatial hearing ability is perturbed on the deaf side. Some monaural listeners learn to adapt the spectral cues, as well as loudness differences created by the acoustic head-shadow, to infer where an object is in space [37,39]. While this does not lead to high accuracy or normal hearing performance, it does allow some lateralization of the signal for reliable loudness cues. However, the monaural cue is not sufficient in providing spatial perception in complex acoustic scenes.

Asymmetry in hearing has been reported to result in a reduced quality of life comparable to [12], or exceeding [40], binaural hearing loss. The combined effect of a reduced ability to orient and reliably extract meaning from speech is particularly deleterious. The binaural summation of signals can increase the perceived loudness of the signal by up to a ratio of 2:1 [41]. In those with SSD, an increase in signal of 3–10 dB is required to obtain the same perceptual improvement [42,43,44]. As a result, there is an increased cognitive load required to selectively attend to a target talker. A lack of contextual information or an ambiguity in speech signals requires an increase in attention to fill in the missing gaps, while a lack of a priori knowledge of to what or where to listen creates uncertainty. In increasingly challenging listening environments where the signal to noise ratio is poor, monaural listeners may not even be aware of a talker positioned at the deaf ear. The behavioral manifestation of this deficit presents as distracted, aloof or inattentive. There is a constant need to adapt and modify to optimally position the good ear for hearing, placing increased demand on the listener. The adverse outcomes associated with SSD, both functional and psychological, should not be underestimated.

## 4. Treatment

Profound sensorineural unilateral hearing loss that is sudden in onset should receive swift medical work-up. Depending on etiology, sudden hearing loss may also be accompanied by ringing in the ear and vestibular symptoms. Where indicated, imaging studies should be considered to rule out retrocochlear pathology as the origin of hearing loss. In some cases, intratympanic or oral steroids can result in a partial or even full recovery of hearing, but the success of such interventions is time dependent [45]. Furthermore, the more severe the loss and the older the patient, the poorer the prognosis for hearing recovery [46,47]. For SSD that cannot be medically managed, there are an increasing number of rehabilitative options available to patients.

### 4.1. Rerouting Solutions

**Figure 4:**
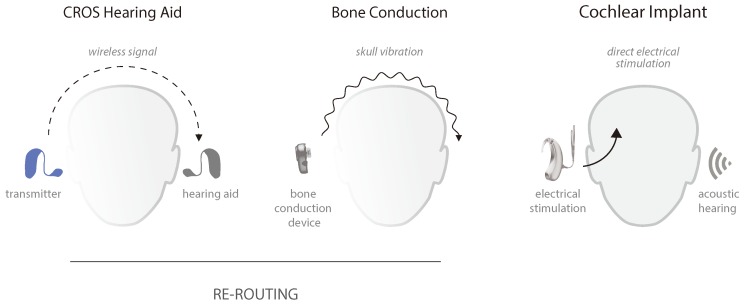
Actual treatment solutions for profound unilateral hearing loss. Rerouting devices transmit the captured sound on the deaf side to the hearing ear (monaural input). In contralateral routing of signal (CROS) hearing aids, the transmitter wirelessly communicates to the contralateral hearing aid. Bone conduction devices transfer the sounds via transcranial skull vibrations towards the functional cochlea. The cochlear implant provides electrical stimulation directly to the deaf ear, preserving the contralateral acoustic hearing (bilateral input).

Traditional hearing rehabilitative options have relied on rerouting the acoustic signal from the deafened ear to the normal or better hearing ear for processing. Contralateral routing of signal (CROS) hearing aids have long been used to reroute the signal of interest to the better hearing ear. This is achieved by placing a microphone behind or in the deaf ear to collect the sound and transmit it wirelessly to a receiver worn in the better ear (Figure 4). CROS hearing aids offer a non-invasive approach to improving access to sound arriving from the deaf side for individuals with SSD. However, access can be an issue for many patients due to the associated high cost and inconsistent coverage for hearing aids globally. The need to wear a receiver in the normal hearing ear may also prove prohibitive, as it disrupts the natural acoustics of the ear, potentially reducing access to some sounds or modifying the monaural pinna cues that unilateral listeners become reliant on to help with directional hearing and the filtering of sounds [48,49]. Osseointegrated bone conduction implants provide an alternative to this, where a microphone on an external sound processor collects the sound and transmits it either percutaneously or transcutaneously (Figure 4) to an osseointegrated implant in the temporal bone of the deafened ear. The signal is then sent via the bones of the skull, where it travels transcranially to directly stimulate the normal hearing inner ear via bone conduction. This allows for the normal hearing ear to stay open and unoccluded, thereby maintaining the monaural spectral cues. In addition to requiring surgery, a limitation of bone conduction stimulation for the purpose of alleviating SSD is the reduction in signal level that may occur as it travels across the skull. This most significantly impacts high frequency cues and is even more prominent under transcutaneous (non-direct) stimulation of the bone.

Rerouting primarily benefits the unilateral listeners by reducing the negative effects of the acoustic head-shadow (Figure 2), by improving the signal-to-noise ratio for deaf ear listening. Studies have demonstrated significant gains in speech understanding under adverse listening when speech is directed at the deaf ear and spatially separated from the noise [35,50,51,52]. However, hearing is negatively affected when noise is directed at the deaf ear and amplified to the normal ear [50]. Importantly, these treatment options are unable to provide any binaural hearing benefits, meaning the unmasking of speech co-located with interfering noise and localization remains largely impaired [50,53].

### 4.2. Cochlear Implantation

Cochlear implants (CI) are an emerging treatment option for SSD (the use of cochlear implants for specific labeling/indications varies by region, and may not be approved or may be subject to restrictions by region), providing direct electrical stimulation to the deafened ear via an electrode placed in the impaired cochlea. Because they provide direct input to the impaired ear, independent stimulation of each ear may provide some binaural hearing benefits not realized with rerouting solutions. Studies of performance in individuals with SSD consistently report improved speech perception in the deaf ear, hearing in noise and localization compared to untreated [54,55,56,57,58] or rerouting conditions [59]. Because CI stimulation is direct to the impaired ear, noise transfer that occurs in rerouting solutions does not impact speech perception in the normal ear. Outcomes, however, continue to deviate from normal hearing performance and are subject to a large degree of variability [54,55,56,59,60]. Speech perception abilities do not match the normal hearing ear, particularly in noise [59,60,61,62], and while significantly improved over rerouting, localization continues to deviate from normal [58,61,62,63]. This localization improvement may be reflective of a lateralization from the loudness cue provided by the CI. This cue is suspected to be more reliable than the previously described loudness cue arising from the head-shadow [37], as it is consistently available to the listener, and the difference is not inferred by the normal ear, but independently processed and determined at the level of the brainstem. This is more reflective of bilateral hearing than binaural hearing, as other characteristic binaural functions such as summation and squelch do not appear to be improved by treatment with CI [54,59]. Bernstein et al. [64] demonstrated squelch in SSD + CI listeners, but only under artificial listening scenarios when the target was completely isolated to the acoustic ear. Results demonstrate the potential of SSD + CI listeners to make use of spatial differences to perceptually separate concurrent voices, although limited, and the cues they use remain uncertain. [64,65] ITDs and ILDs can only be used in situations where the information is 1) reliable and 2) maintained along the many processing points in the auditory pathway.

At present, the CI cannot restore access to ITD cues [66]. Although able to take advantage of one normally functioning ear, ITD sensitivity in SSD + CI listeners is poor compared to in normal hearing listeners, and increased thresholds are observed compared to in bilateral CI or bimodal CI listeners [63,67]. Several researchers have shown a consistent benefit in localization performance in terms of a reduction in overall error [61,62,63]. It is likely that SSD listeners who use a CI in the deaf ear are combining the monaural spectral cue with the loudness difference cue [63] to improve this performance, but that localization accuracy still suffers on the side of the CI, which accounts for the gap from normal. Nonetheless, evidence does suggest that this is more reliable and consistent than what is observed in untreated or rerouting-treated unilateral listeners [61,62,63]. However, for any device to truly restore binaural hearing, it must be able to deliver accurate timing and level cues. CIs are subject to significant processing delays, ranging anywhere from 5 to 20 ms [68]. While such a delay may not significantly impact speech processing, it may inhibit the extent to which information provided through the CI may be used to facilitate binaural hearing abilities. However, others have suggested that direct electrical stimulation overcomes timing delays in the processor to produce shorter timing differences in the brainstem [69]. It remains unclear how these variables in acoustic and electric hearing contribute to binaural processing in constantly and dynamically changing acoustic environments [70]. While CIs are promising, the deviation from normal processing persists in functional outcomes for monaural listeners using a CI. CIs provide a direct stimulation of the deafened ear, which for many listeners results in an improvement in speech perception in the deafened ear [54,55,59,65]. Interestingly, recent findings also suggest that the use of a CI in monaural listening could lead to contralateral speech interference [71]. This negative effect appears to be significantly related to the listener’s age, where older monaural listeners using a CI can experience higher deficits in selective attention. In other words, contralateral speech interference could potentially reduce the before-mentioned benefits provided by the CI in this population, increasing the performance gap from normal hearing listeners.

In traditional CI candidates, the duration of deafness has been shown to be an important predictive factor of hearing outcomes and benefit [72,73,74]. Specifically, a longer duration of deafness is associated with poorer outcomes with CIs. Developing evidence on the impact of the duration of deafness in unilaterally deafened adults suggests similar trends [75]. Of particular relevance is the provision of CIs for children with SSD. Auditory deprivation negatively affects outcomes with CI, leading to global recognition of the importance of early intervention during the critical auditory development periods [76,77,78,79]. Rerouting solutions have limited utility in pediatric populations, and in most cases are not capable of being readily implemented until after the age of five, when most auditory development has already taken place. Unlike rerouting, CI provides stimulation of both auditory pathways. CIs may serve as an important and beneficial alternative, allowing for early intervention by providing additional cues during the critical development phase and before auditory deprivation sets in [77,79,80]. Emerging evidence suggests that the critical period for intervention may play an important role in the long-term outcomes and success of CI in children with SSD [76,77,78,79]. This is supported by animal models pointing to the negative consequences of untreated asymmetric hearing in auditory development, and the ability to restore the binaural auditory system when electrical stimulation is combined with training procedures [81]. However, there are no systematic studies of this in the pediatric population. Current SSD + CI studies vary in terms of the onset of hearing loss, the duration of deafness, and the age of implantation with variable performance outcomes. At present, evidence must be extrapolated from studies of adults with SSD, which are still developing. As an emerging treatment in the SSD population, longitudinal studies are lacking. It’s relevant to mention that in a study of 21 children with congenital SSD, Thomas et al. reported that in those with more than 3 years of follow-up, 3/5 children were limited users or non-users of their CI [82]. This information becomes crucial for practical clinical decisions, cost-effective treatments and patient/parent counseling.

## 5. Summary

Poor spatial hearing, characterized by impaired localization and speech perception in noise, is a common sequela of profound UHL. Much has been learned about the importance of spatial hearing for functional listening abilities. The functional deficits associated with the loss of access to binaural hearing cues have a cascading effect on the unilaterally impaired listener, with high reports of handicap and disability. Evidence suggests that treatments, which reroute the signal from the impaired ear to the normal ear, are successful at reducing the negative effects of the acoustic head-shadow and improving hearing in noise when the signal of interest is located at the deaf side, but are not successful in restoring access to binaural cues. Evidence suggests that the electrical stimulation of the deafened ear by a CI may provide some increased access to these cues, but does not result in the full restoration of binaural hearing abilities. Longitudinal studies are necessary to see how CIs will evolve as treatment solutions in this population. New and/or evolving treatment approaches will be necessary to restore binaural hearing abilities in unilaterally deafened individuals.
